# A two-phase evaluation system integrating hydroponic and field screening identifies nutrient-efficient sweetpotato (*Ipomoea batatas* (L.) Lam.) germplasm

**DOI:** 10.3389/fpls.2026.1780928

**Published:** 2026-02-25

**Authors:** Kang Du, Genmin Lyu, Yongxian Chen, Shunjie Zhang, Qiuming Ye, Pan Pan, Chaobin Yang, Daobin Tang, Jichun Wang, Changwen Lyu, Bo Xu, Kai Zhang

**Affiliations:** 1 College of Agronomy and Biotechnology, Southwest University, Chongqing, China; 2 Zhengzhou Tobacco Research Institute of China National Tobacco Corporation, Zhengzhou, China; 3 Hongta Liaoning Tobacco Co. Ltd, Shenyang, China

**Keywords:** germplasm resources, nutrient use efficiency, sweetpotato (*Ipomoea batatas* (L.) Lam.), two-phase evaluation, yield validation

## Abstract

Sweetpotato (*Ipomoea batatas* (L.) Lam.) is a crucial crop for global food security. However, its sustainable production is hindered by low nutrient use efficiency. Reliable screening protocols that accurately identify nutrient-efficient germplasm of this crop across developmental stages are still lacking. To bridge this gap, we established a novel two-phase evaluation system integrating hydroponic seedling screening with multi-nutrient field validation. We conducted principal component and regression analyses of 35 germplasms lines under controlled deficiencies of nitrogen (N), phosphorus (P), and potassium (K). Five conserved seedling traits were identified, including leaf number per plant, shoot fresh weight, root fresh weight, shoot dry weight, and net photosynthetic rate (Pn). These traits consistently correlated with tolerance to N, P, or K deficiency, thereby supporting their utility as reliable early indicators of nutrient stress. Field validation further confirmed that storage root fresh and dry weight, nutrient content, accumulation, and use efficiency varied significantly among nutrient treatments and genotypes, serving as key indicators of field performance. This integrated approach successfully identified elite germplasm with specific nutrient use efficiency: XN1985–7 as a low-N-tolerant and N-efficient utilization genotype, XN17104–132 as low-K-tolerant and K-efficient utilization, XN2141–3 as low-P-tolerant and P-efficient utilization, and notably XN2153-5, which exhibited concurrent tolerance to low N, P, and K with broad-spectrum efficiency. Our integrated two-phase framework provides a scalable model for screening nutrient-efficient germplasm in root crops, thereby contributing to sustainable breeding programs.

## 1 Introduction

Soil nutrients are critical determinants of land productivity and sustainable agricultural systems (Zhu X.J, et al., 2024). In modern agricultural practices, fertilizer application significantly enhances crop yields (Du et al., 2024; Liao et al., 2023), with data from the FAO indicating that chemical fertilizers account for 50-60% of global food production (Yang et al., 2023). However, excessive use of fertilizers has led to severe environmental consequences, including non-point source pollution, soil acidification, eutrophication, and nutrient loss, all of which contribute to the degradation of soil quality (Cui et al., 2020; Guo et al., 2010; Zhu X.J, et al., 2024). Recent studies estimate that 33% of global soils exhibit moderate to severe degradation, with 52% of agricultural lands significantly affected (Smith et al., 2024). To address these challenges, it is essential to enhance nutrient use efficiency while simultaneously reducing fertilizer inputs. A promising approach involves screening crop cultivars that demonstrate high nutrient use efficiency and maintain stable yields under low-input conditions (Wang et al., 2025).

Sweetpotato is a versatile crop that plays a critical role in food security, livestock nutrition, and industrial processing (Xu et al., 2024). Beyond its importance as a staple food, sweetpotato serves as a valuable vegetable, consumed for its tender leaves and storage roots. Additionally, it plays a role in diversified horticultural systems and is valued as an ornamental plant due to its attractive vines and foliage, underscoring its significance in horticultural production. Sweet potato is cultivated in over 100 countries. Its adaptability to marginal soils, high yield stability (Sapakhova et al., 2024; Shekhar et al., 2015), and nutrient-dense profile (Ivane et al., 2024), make it an essential crop for smallholder farmers. China dominates global sweetpotato production, contributing 48.5% of worldwide output, amounting to 51.4 million tons in 2023 (FAO, 2024). However, this staple crop faces a significant sustainability challenge. Despite a 28% increase in nitrogen (N) fertilizer use since 2000 (FAO, 2022), its N utilization efficiency remains markedly lower than that of cereals. This gap is attributed to its unique asynchronous nutrient requirements, which prioritize N during vegetative growth but shift toward phosphorus (P) and (K) as dominant requirements during storage root bulking (Bourke, 1985). This temporal mismatch in nutrient demand dynamics, unlike that of graminaceous species with synchronized nutrient uptake, not only reduces nutrient use efficiency but also exacerbates leaching losses, thereby driving soil degradation.

Therefore, accelerating the development of nutrient-efficient germplasm is imperative to balance productivity with ecological sustainability. The green development of agriculture emphasizes the reduction of resource consumption and environmental pollution while ensuring food security. Efficient and sustainable nutrient utilization in crops is essential for maintaining soil health, crop yields, and ecosystem balance. Current efforts to address these challenges focus on genotypic screening under nutrient stress. Wang et al. (2015) systematically evaluated seven genotypes under varying K regimes, categorizing them into high, moderate, and low efficiency groups based on K uptake efficiency, K accumulation value, and storage root dry matter yield. Genotypes such as Nan88 and Xu082 exhibited high yield potential with moderate K responsiveness, while Wan5 and Xu28 demonstrated superior use efficiency. Building on this research, Liu et al. (2023) screened 216 sweetpotato genotypes under different K availability conditions, identifying shoot dry matter accumulation and total K content as key indicators for selecting low-K tolerance genotypes, with Jizishu 18 and Guangzishu 2 showing particular adaptability. Similarly, Zhu et al. (2023) analyzed 63 genotypes under P stress, revealing that Xushu 34 and Tanzania exhibited exceptional performance in nutrient-limited environments. Earlier classifications by George et al. (2002) based on K efficiency ratios and root yield responses further identified Nanshu 88 and Xushu 18 as high-efficiency genotypes. Collectively, these studies have established valuable preliminary screening methods and identified promising germplasm.

Notably, sweetpotato exhibits stage-specific nutrient demands and utilization patterns (Lv and Lu, 2021). However, current research predominantly evaluates nutrient responses in isolation—such as K or P efficiency—using controlled hydroponic systems at the seedling-stage (Chen and Liao, 2017; Xia et al., 2025). While these approaches provide foundational insights, they inadequately address two critical processes in the field processes: the interaction of sweetpotato root exudates with field soil, which regulates nutrient acquisition, and the contrasting nutrient allocation priorities between early growth stages, characterized by nitrogen-dependent root establishment, and late field stages, driven by K for storage root yield. These knowledge gaps impair the screening of nutrient-efficient germplasms and compromise the accuracy of evaluation criteria under diverse agronomic conditions. To date, reliable screening protocols for identifying nutrient-efficient germplasm across different growth stages remain underdeveloped in sweetpotato. Integrating hydroponic screening during the seedling stage with field validation throughout the entire growth cycle may address the limitations of single-method or single-stage approaches. This strategy is not only necessary but also efficient for screening nutrient-efficient genotypes in sweetpotato, as it shortens the screening cycle and improves accuracy.

To address the critical gaps in root-soil interaction dynamics and stage-specific nutrient allocation, this study aimed to establish a reliable two-phase screening protocol that integrates hydroponic seedling evaluation with field validation throughout the entire growth cycle. To this end, we applied this two-phase system to assess 35 sweetpotato germplasms. Through comprehensive analyses of growth, nutrient uptake efficiency, and yield performance under stress conditions, we aimed to: (1) identify key physiological and agronomic indicators of nutrient stress tolerance and utilization efficiency; and (2) select superior genotypes exhibiting proven resilience and high nutrient use efficiency. The identified nutrient-efficient germplasms provide essential breeding materials for the development of cultivars suited to low-fertility soils. Furthermore, the established evaluation criteria offer a robust methodological framework for screening nutrient-efficient germplasm across horticultural crops, thereby advancing both practical sweetpotato breeding and the theoretical foundations for sustainable crop improvement.

## 2 Materials and methods

### 2.1 Experiment location and plant materials

#### 2.1.1 Preliminary hydroponic screening experiment

A preliminary hydroponic screening experiment was conducted in 2022 to evaluate nutrient-use efficiency of 35 sweetpotato genotypes and to identify candidates for subsequent studies. The plant materials consisted of 35 sweetpotato germplasm resources (**Supplementary Table S1**), which exhibited divergent morphological and agronomic traits, provided by the Key Laboratory of Potato Biology and Genetic Breeding of Chongqing Municipality. Uniform vine cuttings (30 cm in length) were established in a hydroponic system. Four nutrient treatments were established based on prior studies (Deng et al., 2020; Li et al., 2014a; Sun et al., 2021) with slight modifications: (1) Control (CK) with sufficient supply of N, P, and K; (2) Low N (LN, 0 mmol L^–1^ NO_3_^-^); (3) Low P (LP, 0 mmol L^–1^ PO_4_^3-^); (4) Low K (LK, 0 mmol L^–1^ K^+^). The composition of the full-strength nutrient solution for CK was detailed in **Supplementary Table S2**. The LN, LP, and LK treatments followed a randomized complete block design with three replications, each containing two plants per genotype. During the establishment phase, seedlings were pre-acclimatized in distilled water for 10 days to facilitate root initiation and the development of three fully expanded leaves newly formed during the acclimation period. Nutrient stress treatments were subsequently initiated through daily replenishment of the respective solutions each morning (8:00-9:00) by adding solution to maintain the initial 8 cm depth, which were replaced every three days with fresh solution of the same composition to maintain nutrient stability. Each hydroponic container had a volume of 2 L, and the 8 cm depth corresponded to approximately 1.6 L of solution per container. Growth chambers were maintained at 25 ± 1 °C, 70 ± 5% relative humidity, and a 12-hour light/dark cycle with a photosynthetic photon flux density (PPFD) of 250 μmol m^-2^ s^-1^ provided by white LED growth lights. After 28 days of treatment, photosynthetic parameters and agronomic characteristics were measured.

#### 2.1.2 Field trails and advanced screening validation

Based on the comprehensive evaluation value (Y) derived from the hydroponic screening, we selected eight genotypes that represent a broad spectrum of efficiency, ranging from high to low, for field validation in 2023. This selection was made to investigate genotype-specific responses to varying fertilizer levels. The basic soil nutrient properties at a depth of 0–20 cm are presented in **Supplementary Table S3**. A randomized complete block design with three replications was utilized. The six fertilizer treatments included: Low Nitrogen (LN), High Nitrogen (HN), Low Phosphorus (LP), High Phosphorus (HP), Low Potassium (LK), and High Potassium (HK), respectively. This design resulted in a total of 144 experimental plots. Each plot measured 1.8 m², with a plant spacing of 22.5 cm (equivalent to 2,964 plants per 667 m²). Vine cuttings were planted on June 2, 2023. Fertilizers were applied via the hole-application method 10 days after planting, using urea (46% N), calcium superphosphate (12% P_2_O_5_), and potassium sulfate (52% K_2_O). The specific application rates for each treatment are provided in **Supplementary Table S4**. The “high” level (HN, HP, and HK) treatments were set at or slightly above the locally recommended optimum fertilizer rates for sweetpotato production, thereby representing a condition of non-limiting nutrient supply. In contrast, the “low” level (LN, LP, and LK) treatments were designed to induce moderate nutrient stress by applying zero additional N, P, or K fertilizer, respectively, beyond the basal soil background.

### 2.2 Indicators measurement and methods

#### 2.2.1 Measurement of agronomic traits

In the hydroponic screening trial, plant growth traits were measured for each replication. Following established protocols (Wera, 2014; Yildirim et al., 2011), we measured the following traits: main stem length, adventitious root length, leaf number per plant, shoot fresh weight, root fresh weight, shoot dry weight, and shoot dry matter increase. Each measurement was replicated three times and the mean value was used for subsequent analysis. In the field trials, all sweetpotato plants were harvested at the appropriate harvest stage. The fresh weight of storage roots was recorded for each plot to determine yield. Five plants were randomly selected from each plot to assess shoot and the fresh weight of storage root. The shoot and storage root samples were cleaned, sliced, and heated at 105 °C for 30 minutes to deactivate enzymes, followed by drying at 80 °C until a constant weight was achieved. The storage root dry weight (g), shoot dry weight (g) and whole plant dry weight (g) were documented as described by Huang et al. (2020).

#### 2.2.2 Measurement of photosynthetic parameters

Photosynthetic parameters were measured between 9:00 and 11:00 A.M. on the day prior to sampling. On each of three representative plants per plot, the youngest fully expanded leaf was selected for the measurement of the Pn, stomatal conductance (Gs), intercellular carbon dioxide (CO_2_) concentration (Ci), and transpiration rate (Tr), using a LI-6400 portable photosynthesis system (LI-COR Biosciences, Lincoln, NE, USA) following standard procedures (Wellburn, 1994).

#### 2.2.3 Nutrient content measurement

The concentrations of N, P, and K in plant samples were determined after digestion with H_2_SO_4_-H_2_O_2_. Total N in the samples was determined using the sulfuric acid-hydrogen peroxide digestion method in conjunction with an automatic Kjeldahl apparatus (Chen et al., 2016; Du et al., 2024). Total P was quantified by the vanadium-molybdenum yellow colorimetric method using a spectrophotometer (UV-1500, Macylab, Shanghai, China) (Lu, 1999). Total K was analyzed using the sulfuric acid-hydrogen peroxide digestion method with a flame photometer (AA-7000, SHIMADZU, Kyoto, Japan) (Deng et al., 2021). The soil alkaline-N content was determined by the alkali diffusion method described by Chen et al. (2016). Available P and available K were measured using a Top nutrient analyzer (TPY-16A, *Zhejiang Top Cloud-Agri Technology Co., Ltd., China*) following the standard methodology.

#### 2.2.4 Calculation of plant nutrient uptake and use efficiency indices

Nutrient accumulation and utilization parameters were calculated following established methods from previous studies. The formulas employed are outlined below:

N accumulation: N concentration × Dry matter weight (Deng et al., 2021).

N physiological use efficiency (%): Dry matter increase/N accumulation value (Liu et al., 2023).

Root-shoot ratio: Root dry weight/Shoot dry weight (Liu et al., 2023; Tang et al., 2014).

N harvest index: N accumulation in storage root/N accumulation in all organs (Deng et al., 2021).

N uptake efficiency (kg·kg^-^¹): N accumulation in plants/N applied (Deng et al., 2021).

Storage root N utilization efficiency (kg·kg^-^¹): storage root yield (fresh weight)/total plant N accumulation (Hitz et al., 2017).

Plant N utilization efficiency (kg·kg^-^¹): whole-plant yield (fresh weight)/total plant N accumulation (Hitz et al., 2017).

Relative yield: storage root yield of LN/storage root yield of HN (Deng et al., 2021).

N sensitivity index: storage root yield of N treatment/storage root yield of no N treatment (Tang et al., 2014).

Yield reduction rate (%): (storage root yield of HN – storage root yield of LN)/storage root yield of HN × 100%.

N agronomic use efficiency (kg·kg^-^¹): (Yield in N applied area – Yield in non-N applied area)/N applied (Du et al., 2024; Lemma et al., 2023).

N partial factor productivity (kg·kg^-^¹): Yield in N applied area/N application rate (Du et al., 2024).

N fertilizer contribution rate (%): (Yield of N fertilizer application area – Yield of no N fertilizer application area)/Yield of N fertilizer application area × 100% (Wu et al., 2016).

N utilization index and Low-N tolerance index was calculated according to the Group Standard (T/GDSMM0009-2021) as below:

N utilization index: (storage root yield of no N treatment/Average storage root yield of no N treatment) × (storage root yield of N treatment/Average storage root yield of N treatment).

Low-N tolerance index: (Storage root yield of no N treatment/Average storage root yield of no N treatment) × (Storage root yield of no N treatment/Average storage root yield of N treatment).

The calculations of P and K follow the same procedures.

### 2.3 Comprehensive value calculation and classification method of N, P, and K efficiency

To comprehensively evaluate the low-nutrient stress tolerance of the 35 genotypes from the hydroponic screening, 14 measured indicators were subjected to Principal Component Analysis (PCA) for dimensionality reduction. The composite evaluation value (Y) for each genotype was then calculated based on the PCA results. The comprehensive value of nitrogen efficiency Y is defined as *Σ^nj^=_1_*^[^*^U^*^(^*^Xj^*^)^*^× Ej^*^]^, where membership function *U* (*X_j_*) is calculated as (*X_j_*-*X_j_*_min_)/(*X_jmax_*-*X_jmin_*). The weights *E_j_* are determined by *E_j_ =C_j_/Σ^nj^=_1_C_j_*, where *Xj* denotes the *j*th screening indicator, *X_(j)_*_min_ and *X_j_*_max_ represent the minimum and maximum values of the *j*th evaluation index, respectively, and *Cj* represents the coefficient of variation (CV) of the *j*th evaluation index.

The comprehensive values (Y) for all genotypes were ranked, and this ranking served as the primary basis for selecting the eight genotypes for the subsequent field trial. The calculations for P and K efficiency comprehensive values follow the same methodology.

### 2.4 Statistical analysis

All data were subjected to analysis of variance (ANOVA). The significance of differences between treatment means was determined using Fisher’s Least Significant Difference (LSD) test at a significance level of P < 0.05. All ANOVAs were performed using SPSS Statistics software (Version 26.0, IBM Corp., Armonk, NY, USA). Figures were generated using the ggplot2 package in R software (Version 4.3.1). Data visualization and Principal Component Analysis (PCA) were conducted in R utilizing relevant packages, such as factoextra and FactoMineR.

## 3 Results

### 3.1 Screening of comprehensive evaluation indices for low-nutrition tolerance at seedling stage

#### 3.1.1 Variation analysis of each index under different N, P and K levels at seedling stage

Under various nutrient treatments, 35 sweetpotato genotypes exhibited differential responses in seedling phenotypic and physiological indices (**Table 1**). Specific nutrient deficiencies triggered distinct adaptive responses. Compared to the control (CK), the LN treatment significantly increased root length by 15.2%, and enhanced root fresh weight by 9.8%. However, it also resulted in a notable reduction in stomatal conductance by 45.94%. Shoot N physiological use efficiency increased by 22.6% under LN stress. LP conditions enhanced root length by 18.3% but caused a sharp reduction of 69.77% in shoot P accumulation. The LK treatment specifically improved shoot K physiological use efficiency by 19.1%, with a concomitant decrease of 67.4% in Tr.

**Table 1:** Variability in agronomic and physiological indices of sweet potato germplasm seedlings under differential nitrogen (N), phosphorus (P), and potassium (K) regimes.

Agronomic and physiological indices	CK			Low nitrogen (LN)			Low phosphorus (LP)			Low potassium (LK)		
	Scope	Mean value	CV (%)	Scope	Mean value	CV (%)	Scope	average value	CV (%)	Scope	Mean value	CV (%)
Main stem length (cm)	42.25-128.00	82.59	24.51	17.00-144.25	64.13	40.29	32.75-127.75	67.39	32.32	24.00-109.50	68.57	29.46
Root length (cm)	10.25-32.75	22.61	25.17	5.75-52.5	32.65	35.86	12.75-41.75	29.04	28.55	7.25-34.75	20.24	32.17
leaf number per plant (No.)	5.25-19.25	12.56	26.99	2.50-14.75	9.71	28.89	5.50-17.25	10.51	29.03	3.50-17.25	11.11	33.16
Shoot fresh weight (g)	18.41-89.55	45.96	38.25	14.00-64.00	33.70	39.62	14.88-64.60	36.75	37.68	11.59-69.75	34.58	41.32
Root fresh weight (g)	1.38-11.60	4.09	50.99	1.11-11.23	4.22	56.71	1.03-8.03	3.36	49.65	0.81-7.13	3.23	52.01
Shoot dry weight (g)	1.44-9.81	4.19	40.71	1.30-7.16	3.61	37.22	1.44-6.73	3.74	35.30	1.38-7.77	3.69	37.37
Shoot dry matter increase (g)	0.04-5.41	1.62	80.79	0.01-3.19	1.00	94.99	0.10-3.10	1.14	81.20	0.03-3.87	1.17	79.90
Net photosynthetic rate (μmol m^-2^ s^-1^)	3.74-17.01	9.53	29.78	0.83-11.67	7.70	32.81	0.31-16.27	7.35	68.19	0.73-15.75	6.25	60.48
Stomatal conductance (mol H_2_O m^-2^ s^-1^)	0.06-0.56	0.31	39.95	0.01-0.49	0.17	60.70	0.003-0.50	0.15	87.36	0.02-0.57	0.14	99.17
Intercellular CO_2_ concentration (μmol CO_2_ mol^-1^)	284.73-360.16	328.38	5.36	229.59-380.99	306.93	12.50	200.53-357.50	291.84	12.55	214.17-440.54	326.62	20.04
Transpiration rate (mmo m^-2^ s^-1^)	2.50-12.18	7.81	38.61	0.58-8.81	4.40	51.21	0.19-9.889	3.93	73.23	0.72-7.75	2.54	65.80
Shoot N (P/K) content (g kg^-1^)	24.55-46.13	34.26	13.32	16.06-42.36	23.54	23.82	0.51-4.27	2.13	37.62	9.86-19.76	15.43	18.19
Shoot N (P/K) accumulation value (g plant^-1^)	0.58-3.40	1.42	40.64	0.44-1.68	0.82	36.97	0.02-0.15	0.08	43.05	0.24-1.29	0.56	41.75
Shoot N (P/K) physiological use efficiency (%)	0.05-4.03	1.10	69.01	0.02-3.17	1.15	82.10	1.32-8.28	1.59	96.25	0.10-4.97	1.93	59.99

The coefficients of variation (CVs) for all traits, calculated to assess their discriminative power, ranged from 5.36% to 80.79% for CK, 12.50% to 94.99% for LN, 12.55% to 96.25% for LP, and 18.19% to 99.17% for LK. Under all stress conditions, the shoot N, P, and K contents, along with the Ci, exhibited low variability (less than 30%), indicating minimal impacts from nutrient stress. In contrast, traits such as leaf number per plant, root morphology (including root length and fresh weight), shoot biomass (both fresh and dry weight, as well as dry matter increase), photosynthetic parameters (Pn, Gs, and Tr), and nutrient use efficiency indices (N, P, and K accumulation values, physiological use efficiency) showed significant variation, making them effective indicators for assessing nutrient use efficiency in sweetpotato germplasm.

#### 3.1.2 Principal component analysis for integrative evaluation

Given the complexity of nutrient stress responses, PCA was employed to integrate 14 phenotypic and physiological indices. PCA identified four principal components (PCs) with eigenvalues >1 for each nutrient stress (**Table 2**), which collectively explained 83.22%, 82.79%, and 76.19% of the total variance under N, P, and K deficiency, respectively, indicating effective dimensionality reduction.

**Table 2:** PCA results for traits under nutrient deficiency: weighted coefficients, eigenvalues, variance contribution rates, and cumulative contributions.

Agronomic and physiological indices	Principal component (low nitrogen stress)				Principal component (low phosphorus stress)				Principal component (low potassium stress)			
	PC1	PC2	PC3	PC4	PC1	PC2	PC3	PC4	PC1	PC2	PC3	PC4
Main stem length (cm)	0.362	0.135	0.137	-0.459	0.343	-0.113	0.026	-0.038	0.284	-0.168	-0.366	-0.036
Root length (cm)	0.297	-0.265	0.209	0.287	0.124	0.354	0.280	-0.460	0.315	0.015	0.026	-0.249
leaf number per plant	0.355	0.153	0.157	-0.342	0.349	-0.052	0.125	-0.121	0.355	-0.097	-0.379	-0.134
Shoot fresh weight (g)	0.346	0.191	0.023	-0.287	0.368	0.008	-0.093	-0.069	0.376	0.021	-0.332	0.073
Root fresh weight (g)	0.232	-0.055	0.323	0.513	0.189	0.418	0.013	-0.304	0.327	0.140	0.115	-0.052
Shoot dry weight (g)	0.302	0.253	-0.229	0.219	0.356	0.103	0.065	0.029	0.398	-0.111	0.162	-0.060
Shoot dry matter increase (g)	0.332	0.134	-0.262	0.202	0.183	0.445	-0.176	0.406	0.303	-0.232	0.267	0.296
Net photosynthetic rate (μmol CO_2_ m^-2^ s^-1^)	-0.051	0.352	0.396	0.175	0.309	-0.210	-0.179	0.135	0.078	0.457	-0.099	0.378
Stomatal conductance (mol H_2_O·m^-2^ s^-1^)	-0.164	0.405	0.282	0.108	0.303	-0.263	-0.287	-0.047	0.136	0.505	-0.010	0.284
Intercellular CO_2_ concentration (μmol CO_2_ mol^-1^)	-0.137	0.388	-0.072	-0.126	0.227	-0.024	0.158	-0.390	0.030	0.326	-0.409	0.001
Transpiration rate (mmol H_2_O m^-2^ s^-1^)	-0.137	0.401	0.326	0.082	0.255	-0.317	-0.331	-0.060	0.151	0.269	0.366	0.268
Shoot N content (g kg^-1^)	-0.271	0.214	-0.355	-0.006	0.113	-0.162	0.604	0.289	0.016	0.294	0.258	-0.446
Shoot N accumulation value (g plant^-1^)	0.040	0.334	-0.419	0.258	0.260	-0.072	0.442	0.373	0.340	0.125	0.312	-0.354
Shoot N physiological use efficiency (%)	0.371	0.103	-0.195	0.156	0.172	0.473	-0.226	0.332	0.176	-0.361	0.159	0.447
Eigenvalue (math.)	4.619	4.071	1.894	1.067	6.128	2.435	1.774	1.254	4.385	3.058	1.752	1.472
Variance contribution (%)	32.995	29.077	13.532	7.619	43.768	17.395	12.675	8.956	31.320	21.842	12.515	10.513
The cumulative contribution rate (%)	32.995	62.072	75.604	83.224	43.768	61.164	73.839	82.794	31.32	53.162	65.676	76.189

In the analysis of N responses, critical positive loadings were identified for shoot N physiological use efficiency (0.371 in PC1), Gs (0.405 in PC2), and root fresh weight (0.513 in PC4). Conversely, the shoot N accumulation value displayed a negative loading (-0.419 in PC3). The components accounted for variance contributions of 33.00% in PC1, 29.08% in PC2, 13.53% in PC3, and 7.62% in PC4, respectively, collectively explaining 83.22% of the variance. This confirms the effectiveness of dimensionality reduction in characterizing N responses.

In the P analyses, significant positive loadings were observed for shoot fresh weight (0.368 in PC1), shoot P physiological use efficiency (0.473 in PC2), and shoot phosphorus content (0.442 in PC3), while a negative loading was noted for root length (-0.460 in PC4). The variance contributions were ranked at 43.77% for PC1, 17.40% for PC2, 12.68% for PC3, and 8.96% for PC4, achieving a cumulative variance of 82.79% and validating the model’s capacity to distill the complexity of P response.

Regarding K assessments, key positive loadings were observed for shoot dry weight (0.398 in PC1), Gs (0.505 in PC2), and shoot K physiological use efficiency (0.447 in PC4), whereas Ci negatively influenced PC3 (-0.409). The component contributions were 31.32% (PC1), 21.84% (PC2), 12.52% (PC3), and 10.51% (PC4), collectively explaining 76.19% of variance and demonstrating a robust extraction of K response patterns.

#### 3.1.3 Regression analysis of sweetpotato seedling stress index and comprehensive evaluation Y value

To establish a simplified model for predicting the comprehensive evaluation value (Y), stepwise regression was performed between Y and the stress tolerance indices of the 14 agronomic traits (**Table 3**). In the N tolerance response, we found that the tolerance indices for eight traits (including main stem length, leaf number per plant, shoot fresh weight, root fresh weight, shoot dry weight, shoot dry matter increase, Pn, and shoot N physiological use efficiency) exhibited highly significant correlations with Y. After addressing multicollinearity by eliminating the shoot dry matter increase, a stepwise regression model was established with the remaining seven traits. The resulting equation Z = 4.121 + 0.541X_1_ + 0.677X_3_ + 0.592X_4_ + 0.366X_5_ + 1.122X_6_ + 1.188X_8_ + 0.164X_14_, showed strong predictive capability with R^2^ = 0.985, where the regression coefficients indicated the contribution weights of the variables. Through comprehensive variation, principal component, and regression analyses, seven key evaluation criteria were identified for N deficiency tolerance at the seedling stage: main stem length, leaf number per plant, shoot fresh weight, root fresh weight, shoot dry weight, net photosynthetic rate, and shoot N use efficiency.

**Table 3:** Correlation analysis of low nutrient tolerance indices with comprehensive evaluation value (Y).

Serial number	Indices	nitrogen treatment	*P*-value	phosphorus treatment	*P*-value	potassium treatment	*P*-value
		Correlation coefficient		Correlation coefficient		Correlation coefficient	
X_1_	Main stem length	0.752**	0.000	0.771**	0.000	0.244	0.157
X_2_	Root length	0.230	0.185	0.435**	0.009	0.52**	0.001
X_3_	Leaf number per plant	0.779**	0.000	0.819**	0.000	0.405*	0.016
X_4_	Shoot fresh weight	0.781**	0.000	0.849**	0.000	0.597**	0.000
X_5_	Root fresh weight	0.438**	0.009	0.576**	0.000	0.711**	0.000
X_6_	Shoot dry weight	0.764**	0.000	0.896**	0.000	0.633**	0.000
X_7_	Shoot dry matter increase	0.652**	0.000	0.604**	0.000	0.464**	0.005
X_8_	Net photosynthetic rate	0.483**	0.003	0.630**	0.000	0.572**	0.000
X_9_	Stomatal conductance	0.330	0.053	0.557**	0.001	0.718**	0.000
X_10_	Intercellular CO_2_ concentration	0.235	0.174	0.520**	0.001	0.216	0.213
X_11_	Transpiration rate	0.377*	0.025	0.414*	0.013	0.647**	0.000
X_12_	Shoot N content	-0.267	0.120	0.356*	0.036	0.263	0.127
X_13_	Shoot N accumulation value	0.392*	0.020	0.712**	0.000	0.716**	0.000
X_14_	Shoot N physiological use efficiency	0.689**	0.000	0.571**	0.000	0.132	0.448

* and ** represent significant levels of difference of 0.05 and 0.01, respectively.

Similar analytical procedures for P deficiency tolerance (**Table 3**) revealed highly significant correlations between yield (Y) and twelve indices, including main stem length, root length, leaf number per plant, shoot fresh weight, root fresh weight, shoot dry weight, shoot dry matter increase, Pn, Gs, Ci, shoot P accumulation, and shoot P physiological use efficiency. Subsequent regression modeling, after addressing multicollinearity effects produced the equation Z = 4.284 + 0.690X_1_ + 0.443X_2_ + 0.735X_3_ + 0.408X_4_ + 0.267X_5_ + 0.286X_6_ + 0.139X_8_ + 0.113X_9_ + 0.642X_10_ + 2.419X_13_ + 0.031X_14_ (R^2^ = 0.999, *P* < 0.001). This analysis ultimately identified ten critical indices for evaluating phosphorus tolerance: main stem length, root length, leaf number per plant, shoot fresh weight, root fresh weight, shoot dry weight, Pn, Gs, shoot P accumulation value, and shoot P physiological use efficiency through multidimensional analysis.

In the assessment of K deficiency (**Table 3**), ten parameters were identified as exhibiting significant importance: root length, leaf number per plant, shoot fresh weight, root fresh weight, shoot dry weight, shoot dry matter increase, Pn, Gs, Tr, and shoot K accumulation. The optimized regression mode, represented by the equation Z = - 3.590 + 0.482X_2_ + 0.191X_3_ + 0.519X_4_ + 0.303X_5_ + 0.318X_6_ + 0.263X_7_ + 0.610X_8_ + 0.780X_9_ + 0.810X _11_ + 1.433X_13_ demonstrated an exceptional fit (R^2^ = 0.999, *P* < 0.001). This analysis enabled the selection of these ten metrics for evaluating K tolerance through integrated analytical approaches.

### 3.2 Screening of germplasm resources for nutrient use efficiency at seedling stage

#### 3.2.1 Cluster analysis of low N, P and K stress tolerance index of sweetpotato at seedling stage

**Figure 1:**
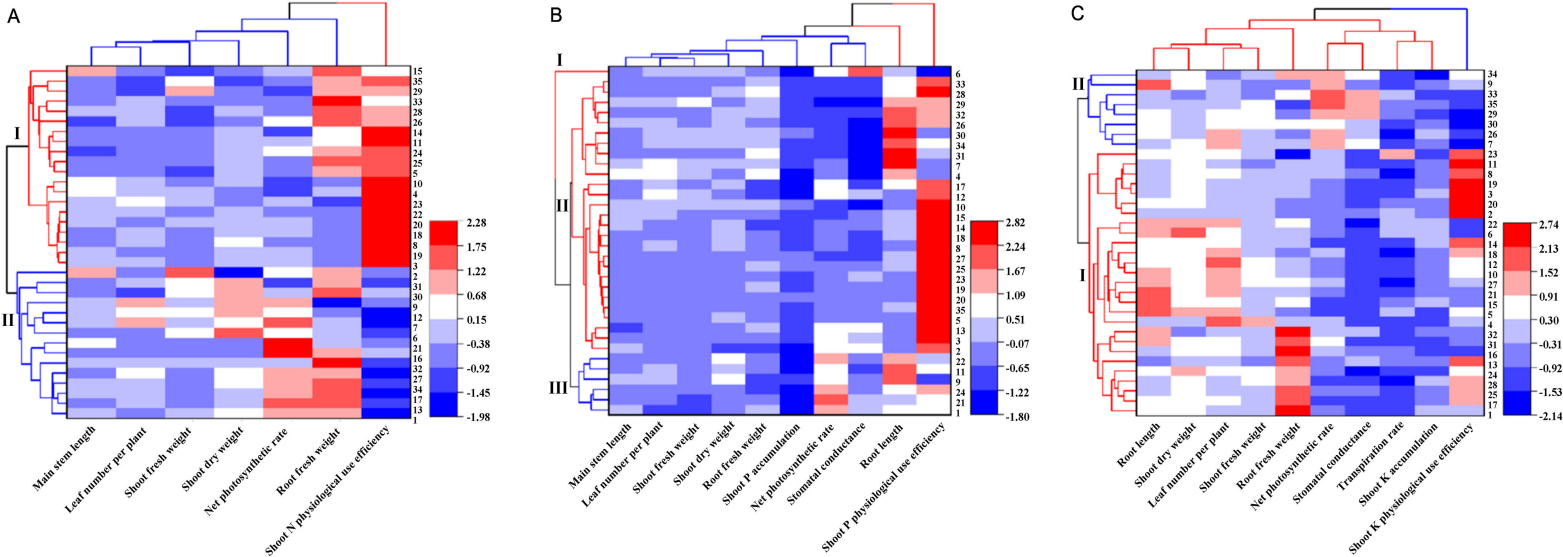
Cluster analysis of 35 genotypes of sweet potato evaluated for tolerance indices under conditions of low nitrogen **(A)**, phosphorus **(B)**, and potassium **(C)**.

Cluster analysis utilizing Euclidean distance was conducted on the evaluation indices for low N, P, and K tolerance, with the results illustrated in **Figure 1**. In the LN assessment (**Figure 1A**), the 35 germplasms were categorized into two clusters. Cluster I, comprising 20 germplasms, characterized by superior root fresh weight and shoot N physiological use efficiency, was identified as low-N tolerant (mean Y = 0.33). Conversely, Cluster II, consisting of 15 germplasms, despite higher shoot dry weight and Pn, was less efficient and classified as low-N sensitive (mean Y = -0.44).

In the LP analysis (**Figure 1B**), three clusters were identified. The majority of germplasms (Clusters I and II, encompassing 29 germplasms), exhibited greater root length and enhanced shoot P use efficiency and were classified as low-P tolerant. In contrast, Cluster III, comprising 6 germplasms, was sensitive to P deficiency. Regarding the LK evaluation (**Figure 1C**), two clusters were formed. Cluster I, consisting of 27 germplasms, demonstrated larger root length, greater shoot dry weight, increased leaf number per plant, higher root fresh weight, and improved N use efficiency, was determined to be the low K-tolerant type. Conversely, Cluster II, comprising 8 germplasms, exhibited a higher Pn and Gs but lower stress tolerance, was sensitive to K deficiency.

#### 3.2.2 Comprehensive evaluation of nutrient use efficiency of sweetpotato at seedling stage

**Figure 2:**
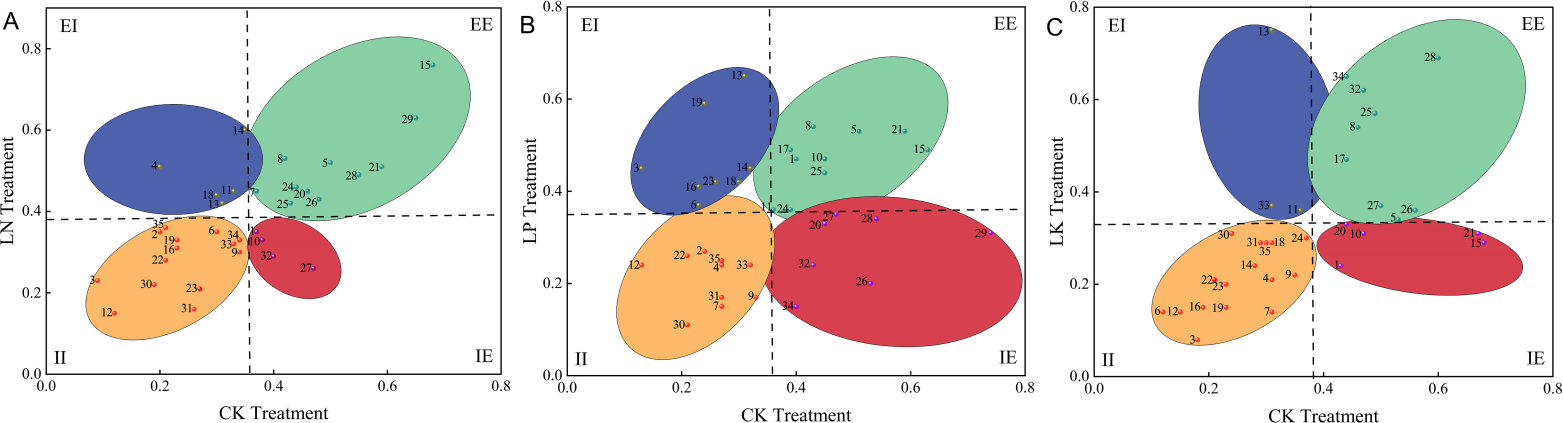
Scatter plots of comprehensive evaluation values for nitrogen **(A)**, phosphorus **(B)**, and potassium **(C)** efficiency of sweet potato germplasms under contrasting nutrient levels. EE, EI, II, and IE indicate Efficient-Efficient, Efficient-Inefficient, Inefficient-Inefficient, and Inefficient-Efficient type, respectively.

Germplasms were further classified into four nutrient use efficiency types based on their performance under CK and stress conditions (**Figure 2**). Comprehensive N efficiency values were calculated under both LN and CK conditions. Scatter plot analysis (**Figure 2A**) categorized the germplasms into four groups: inefficient-efficient (IE type, nutrient-efficient in high nutrient treatment, 11.43%, 4 germplasms), efficient-efficient (EE type, nutrient-efficient in both high and low nutrient treatments, 31.43%, 11 germplasms), inefficient-inefficient (II type, nutrient-efficient in normal or high nutrient treatments, 48.26%, 15 germplasms) and efficient-inefficient (EI type, nutrient-efficient in low nutrient treatment, 14.29%, 5 germplasms).

For P efficiency, indices such as main stem length, root length, leaf number per plant, shoot fresh weight, root fresh weight, shoot dry weight, Pn, Gs, shoot P accumulation value, and shoot P physiological use efficiency were assessed. Under LP and CK treatments with K supplementation, scatter plot analysis (**Figure 2B**) classified the germplasms into EE, (25.71%, 9 germplasms), EI (25.71%, 9 germplasms), II (28.57%, 10 germplasms), and IE (20%, 7 germplasms).

The evaluation of K efficiency evaluation incorporated root length, Tr, and shoot K accumulation value. Scatter plot analysis (**Figure 2C**) under LK and CK treatments revealed EE (25.71%, 9 germplasms), EI (8.57%, 3 germplasms), II (48.57%, 17 germplasms), and IE (14.29%, 5 germplasms). The II category predominated across all nutrients, representing 48.26% for N, 28.57% for P, and 48.57% for K while EE genotypes constituted 25.71 to 31.43% of the population.

#### 3.2.3 Classification of sweetpotato germplasm resources under different N, P, and K levels at the seedling stage

The 35 sweetpotato germplasms were systematically classified into two categories: nutrient stress resilience and nutrient use efficiency types, as detailed in **Supplementary Table S5**. A comprehensive cluster analysis revealed that 13 germplasms exhibited combined tolerance to low N, P, and K stress, while one germplasm demonstrated complete intolerance to low N, P, and K stress, designated as the low N-, P-, and K-intolerant type. Nutrient use efficiency evaluation categorized 4 germplasms as N-, P-, and K-EE types and 5 as N-, P-, and K-II types.

An integrated analysis of low N tolerance indices and N efficiency values identified certain germplasms as low N-tolerant and N-efficient, in contrast to 5 germplasms classified as low N-intolerant and N-inefficient types. A parallel assessment for P utilization distinguished 3 germplasms as low P-tolerant and P-efficient, compared to 5 germplasms identified as low P-intolerant and P-inefficient types. Similarly, K analysis revealed 4 germplasms as low K-tolerant and K-efficient, in comparison to 5 germplasms classified as low K-intolerant and K-inefficient types.

Critical findings identified germplasm XN2153–5 as a low N-, P-, and K-tolerant and highly efficient type, which maintained robust performance across all three nutrient limitations. Conversely, XN2153–1 was characterized as a low N-, P-, and K-intolerant and low-efficient type, demonstrating compromised viability under nutrient stress conditions.

### 3.3 Field validation and screening of nutrient use efficiency

#### 3.3.1 Analysis of agronomic traits and nutrient variation of sweetpotato germplasm under different nutrients in field experiments

Field trials validated and extended the seedling-stage findings. At the harvest stage, the responses of various indices among different sweetpotato germplasm resources under two N levels exhibited discrepancies (**Supplementary Table S6**). The analysis of agronomic traits and nutrient use efficiency-related indices of sweetpotato germplasm resources at the harvest stage revealed that, compared to the HN treatment, the LN treatment resulted in significant declines in most indices, particularly in the storage root N accumulation, storage root N content, storage root fresh weight, N harvest index, shoot dry weight, whole plant dry weight, storage root dry weight, total N accumulation value, total N content, and shoot N accumulation value. This indicated that these traits are highly sensitive to N supply. The root-shoot ratio remained stable across N treatments, demonstrating its insensitivity to N levels. Both storage root N utilization efficiency and plant N utilization efficiency showed increased values under LN conditions, indicating that N limitation triggers adaptive physiological responses that enhance N acquisition and utilization efficiency in sweetpotato. Under LN conditions, the CVs significantly increased for storage root N utilization efficiency, plant N utilization efficiency, root-shoot ratio, shoot N content, storage root N content, total N content, shoot N accumulation value, storage root N accumulation value, total N accumulation value, and N harvest index, indicating that these traits exhibit greater variation among germplasm resources under low N conditions. This reflects enhanced expressivity of genetic variation under N stress and provides an effective screening opportunity for identifying N-efficient genotypes.

The response of various indices to two P levels varied among different sweetpotato germplasms (**Supplementary Table S7**). Compared to the HP treatment, the LP treatment resulted in declines in most indices, particularly the root-shoot ratio, storage root fresh weight, storage root dry weight, storage root P accumulation, total P accumulation, whole plant dry weight, and storage root P use efficiency. This indicated that these biomass-related traits are highly sensitive to P availability. Conversely, shoot P content, total P content, shoot P accumulation, storage root P accumulation, and P harvest index remained relatively stable across P treatments, demonstrating their insensitivity to P levels. Notably, storage root P content exhibited increased values under LP conditions. Under LP conditions, the CVs highly increased for storage root dry weight, P harvest index, storage root P content, shoot P content, total P content, and storage root P utilization efficiency, compared to HP. This suggests that these traits exhibit greater variation among germplasm resources under low P conditions and could serve as critical indices for selecting P-efficient germplasm resources.

The responses of various indices among different sweetpotato germplasms to two K levels also exhibited significant variation (**Supplementary Table S8**). Compared to the HK treatment, the LK treatment resulted in declines in most indices, such as storage root K accumulation, storage root dry weight, storage root fresh weight, root-shoot ratio, storage root K use efficiency, K harvest index, whole plant dry weight, shoot K content, storage root K content, total K accumulation, and plant K utilization efficiency. This indicated that these traits are highly sensitive to K supply. Interestingly, shoot dry weight and shoot K accumulation increased under LK conditions, demonstrating the plant’s adaptive physiological strategies through regulating resource allocation and enhancing nutrient utilization efficiency. Under LK conditions, CVs among germplasms increased for most of the tested indices, with a dramatic increase in root-shoot ratio, K harvest index, total K accumulation, shoot K accumulation, whole plant dry weight, shoot K content, storage root K content, and storage root K utilization efficiency. This suggests that these traits exhibit greater variation among germplasm resources under low K conditions, providing critical targets for selecting K-efficient genotypes. Furthermore, the tested agronomic traits and nutrient use efficiency-related indices varied distinctly under LN, LP, and LK stress, indicating that sweetpotato exhibited different adaptive physiological strategies under different nutrient stress.

#### 3.3.2 Correlation of nutrient use efficiency indexes of sweetpotato plants

**Figure 3:**
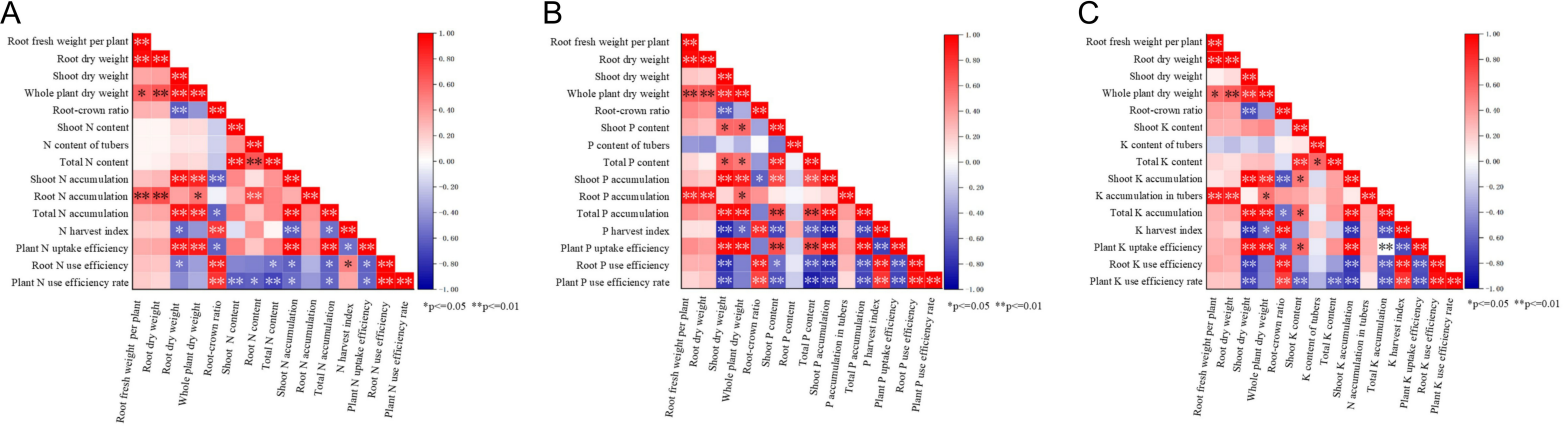
Correlation matrices of agronomic and physiological indicators under low and high levels of nitrogen **(A)**, phosphorus **(B)** and potassium **(C)** treatments. *p < 0.05; **p < 0.01.

Correlation analysis revealed conserved relationships among traits across nutrients (**Figure 3**). Under N treatments (**Figure 3A**), biomass traits exhibited a strong association with N accumulation. Both storage root fresh weight and dry weight demonstrated highly significant positive correlations (*P* < 0.01) with storage root N accumulation. Similarly, shoot dry weight and whole plant dry weight revealed strong positive correlations with shoot N accumulation, total N accumulation, and plant N uptake efficiency. A notable finding was the significant negative correlation (*P* < 0.01) between plant N utilization efficiency and both total N content and shoot N content. Furthermore, storage root N accumulation was positively correlated with both plant N uptake efficiency and plant N utilization efficiency.

Under P treatments (**Figure 3B**), a similar pattern emerged where biomass and accumulation were closely linked. The storage root fresh weight exhibited a positive correlation with storage root P accumulation. Additionally, the shoot dry weight and the whole plant dry weight demonstrated significant positive correlations with the shoot P accumulation as well as the total P accumulation. Root-shoot ratio demonstrated significant positive correlations with P harvest index, storage root P utilization efficiency and plant P utilization efficiency. Moreover, total P content showed a positive correlation with shoot P accumulation and total P accumulation, while exhibiting a highly significant negative correlation with the P harvest index, storage root P utilization efficiency and plant P utilization efficiency. Furthermore, shoot P accumulation and total P accumulation revealed highly significant negative associations with P harvest index, storage root P use efficiency and plant P use efficiency. Storage root P use efficiency and plant P use efficiency exhibited highly significant positive correlations with P harvest value.

In the context of K conditions (**Figure 3C**), storage root fresh weight, storage root dry weight maintained highly significant positive correlations with storage root K accumulation and total K accumulation. Root-shoot ratio demonstrated highly positive correlations with K harvest index, storage root K utilization efficiency and plant K utilization efficiency, while showing negative correlations with shoot K accumulation value. Shoot K content and total K content demonstrated significant negative correlations with plant K utilization efficiency. Shoot K accumulation value and total K accumulation value exhibited significant negative correlations with K harvest index, storage root K utilization efficiency and plant K utilization efficiency. Additionally, K harvest index demonstrated highly significant positive correlations with storage root K utilization efficiency and plant K utilization efficiency.

#### 3.3.3 Field screening of nutrient use efficiency of sweetpotato based on yield

To enhance the robustness of the field validation, eight selected germplasms were incorporated from the initial screen. Field screening revealed an average yield reduction of 19.74% under LN (**Table 4**). Based on the yields of germplasms under HN, LN and their average value, the germplasms could be classified into four categories: EE type, including XN1985–7 and XN1878-7, which yield exceeded the average yield under both HN and LN; EI type, including XN2154–1 and XN2155-1, which yield exceeded the average yield under LN, but lower than the average yield under HN; II type, including XN17104–132 and XN2153-1, which yield was lower than the average both under HN and LN; IE type, including XN2153–5 and XN2141-3, which yield was higher than the average under HN but lower than the average under LN. Notably, XN1878–7 exhibited a superior N sensitivity index, yield increase rate, and agronomic use efficiency, thereby confirming its classification as low N-tolerant and high N- efficient type. Conversely, XN2153–1 was identified as low N-sensitive and inefficient germplasm.

**Table 4:** Yield performance and nitrogen use efficiency indices under low and high nitrogen treatments.

Germplasm name	Low nitrogen (kg ha^-1^)	High nitrogen (kg ha^-1^)	Relative yield	Nitrogen sensitivity index	Yield reduction rate (%)	Nitrogen agronomic use efficiency (kg kg^-1^)	Nitrogen partial factor productivity (kg kg^-1^)	Nitrogen utilization index	Low nitrogen tolerance index	Nitrogen fertilizer contribution rate (%)
XN2153-1	500.00 ± 14.14f	869.33 ± 3.77d	0.58	1.74	42.48	30.78	72.44	0.57	0.33	42.48
XN2153-5	684.00 ± 5.66d	1138.00 ± 2.83b	0.60	1.66	39.89	37.83	94.83	1.01	0.61	39.89
XN1985-7	1146.67 ± 4.71a	1490.83 ± 34.18a	0.77	1.30	23.09	28.68	124.24	2.22	1.71	23.09
XN17104-132	566.43 ± 33.34e	693.33 ± 56.57f	0.82	1.22	18.30	10.58	57.78	0.51	0.42	18.30
XN2154-1	902.50 ± 53.03b	766.50 ± 23.33e	1.18	0.85	-17.74	-11.33	63.88	0.90	1.06	-17.74
XN2155-1	817.67 ± 20.27c	631.67 ± 44.78g	1.29	0.77	-29.45	-15.50	52.64	0.67	0.87	-29.45
XN1878-7	785.00 ± 96.64c	1446.57 ± 71.52a	0.54	1.84	45.73	55.13	120.55	1.48	0.80	45.73
XN2141-3	677.33 ± 15.08d	1051.90 ± 35.69c	0.64	1.55	35.61	31.21	87.66	0.93	0.60	35.61
average value	759.95	1011.02	0.80	1.37	19.74	20.92	84.25	1.04	0.80	19.74

The data is the mean value of three replicates. Different lowercase letters after the same column of data indicate the differences between treatments reach a significant level of 0.05%.

Field trials (**Table 5**) indicated an average yield decline of 16.98% under LP conditions. Likewise, the germplasms could be categorized as EE (XN2153-5, XN2141-3), EI (XN2153-1, XN2155-1), II (XN17104-132, XN2154-1), and IE (XN1878-7, XN1985-7) types. Notably, XN1985–7 exhibited the highest values across all P efficiency indices, including relative value, P sensitivity index, yield increase rate, P agronomic use efficiency and P contribution rate. Integrated analysis of both seedling-stage and field experiments identified two germplasms, XN2153–5 and XN2141-3, which demonstrated superior low P tolerance and high P efficiency, rendering them ideal germplasm resources for investigating P utilization mechanisms in sweetpotato.

**Table 5:** Yield performance and phosphorus efficiency indices under low and high phosphorus treatments.

Germplasm name	Low phosphorus (kg ha^-1^)	High phosphorus (kg ha^-1^)	Relative yield	Phosphorus sensitivity index	Yield reduction rate (%)	Phosphorus agronomic use efficiency (kg kg^-1^)	Phosphorus partial factor productivity (kg kg^-1^)	Phosphorus utilization index	Low phosphorus tolerance index	Phosphorus fertilizer contribution rate (%)
XN2153-1	800.00 ± 82.02cd	967.50 ± 45.96e	0.83	1.21	17.31	20.94	120.94	0.95	0.79	17.31
XN2153-5	1240.00 ± 56.57a	1162.50 ± 53.03c	1.07	0.94	-6.67	-9.69	145.31	1.77	1.89	-6.67
XN1985-7	660.00 ± 16.97e	1650.00 ± 70.71a	0.40	2.50	60.00	123.75	206.25	1.34	0.53	60.00
XN17104-132	763.33 ± 33.00d	701.67 ± 30.64g	1.09	0.92	-8.79	-7.71	87.71	0.66	0.72	-8.79
XN2154-1	336.67 ± 51.85f	820.00 ± 84.85f	0.41	2.44	58.94	60.42	102.50	0.34	0.14	58.94
XN2155-1	863.33 ± 4.71bc	559.64 ± 20.71h	1.54	0.65	-54.27	-37.96	69.96	0.59	0.92	-54.27
XN1878-7	653.33 ± 75.42e	1425.00 ± 63.64b	0.46	2.18	54.15	96.46	178.13	1.14	0.52	54.15
XN2141-3	914.57 ± 81.22b	1077.71 ± 37.17d	0.85	1.18	15.14	20.39	134.71	1.21	1.03	15.14
average value	778.90	1045.50	0.83	1.50	16.98	33.32	130.69	1.00	0.82	16.98

The data is the mean value of three replicates. Different lowercase letters after the same column of data indicate the differences between treatments reach a significant level of 0.05%.

K treatments (**Table 6**) indicated an average of 8.97% reduction in yield under LK conditions. The germplasms also could be categorized into four groups: EE (XN2153-5, XN17104-132), EI (XN1985-7), II (XN1878-7, XN2154-1, XN2155-1, XN2153-1), and IE (XN2141-3). XN17104–132 demonstrated the highest values across K efficiency indices, including K agronomic use efficiency, K partial factor productivity, K utilization index, and low K tolerance index. An integrated analysis of hydroponic and field experiments identified XN2153–5 as a low K-tolerant and K-efficient germplasm, while XN2153–1 was characterized as a low K sensitive and K-inefficient genotype. These contrasting phenotypes provide ideal materials for deciphering K utilization mechanisms in sweetpotato.

**Table 6:** Yield performance and potassium efficiency indices under low and high potassium treatments.

Germplasm name	Low potassium (kg ha^-1^)	High potassium (kg ha^-1^)	Relative yield	Potassium sensitivity index	Yield reduction rate (%)	Potassium agronomic use efficiency (kg kg^-1^)	Potassium partial factor productivity (kg kg^-1^)	Potassium utilization index	Low-potassium tolerance index	Potassium fertilizer contribution rate (%)
XN2153-1	479.50 ± 13.44d	767.50 ± 24.75g	0.62	1.60	37.52	18.00	47.97	0.31	0.19	37.52
XN2153-5	1164.00 ± 50.91b	1544.29 ± 62.63b	0.75	1.33	24.63	23.77	96.52	1.52	1.15	24.63
XN1985-7	1090.00 ± 70.71b	970.00 ± 42.43e	1.12	0.89	-12.37	-7.50	60.63	0.90	1.01	-12.37
XN17104-132	1845.00 ± 49.50a	2333.50 ± 12.02a	0.79	1.26	20.93	30.53	145.84	3.64	2.88	20.93
XN2154-1	553.33 ± 75.42d	844.00 ± 5.66f	0.66	1.53	34.44	18.17	52.75	0.40	0.26	34.44
XN2155-1	943.33 ± 51.85c	582.33 ± 24.98h	1.62	0.62	-61.99	-22.56	36.40	0.47	0.75	-61.99
XN1878-7	994.67 ± 82.97c	1123.75 ± 5.30d	0.89	1.13	11.49	8.07	70.23	0.95	0.84	11.49
XN2141-3	998.39 ± 79.04c	1205.00 ± 7.07c	0.83	1.21	17.15	12.91	75.31	1.02	0.84	17.15
average value	1008.53	1171.30	0.91	1.20	8.97	10.17	73.21	1.15	0.99	8.97

The data is the mean value of three replicates. Different lowercase letters after the same column of data indicate the differences between treatments reach a significant level of 0.05%.

#### 3.3.4 Identification of nutrient use efficiency germplasms based on two-phase evaluation system

Comprehensive analysis of the two phases (hydroponic screening and field trial) results revealed significant differentiation in nutrient utilization characteristics among the tested germplasm resources (**Supplementary Table S9**). XN2153–5 showed tolerance to low N, P, and K conditions at the seedling stage, and its good yield performance under LN, LP and LK also confirmed its low-N/P/K tolerance. XN1985–7 was identified as N-efficient type during two phases and could be identified as low N- tolerant and N-efficient genotype. XN2141–3 showed N/P/K-tolerance at seedling stage, and P-efficiency under the two phases, suggesting it’s a low P-tolerant and P-efficient genotype. XN17104–132 showed N/P-inefficiency but exhibited low K-tolerance and efficiency, and showed the highest K agronomic use efficiency, partial factor productivity, utilization index, and low-K tolerance index, and could be identified as a K-efficient genotype.

XN2153–1 was identified as a low N-, P-, and K-intolerant and II genotype during the seedling stage, and field experiments further confirmed its low N/P/K utilization index and low-N/P/K tolerance index. Therefore, XN2153–1 could be identified as a low N-, P-, and K-intolerant and -inefficient germplasm. In contrast, XN2153–5 exhibited outstanding comprehensive traits. This germplasm was consistently classified as a low N-, P-, and K-tolerant and EE type at the seedling stage. Particularly under LP conditions, its yield reached 1240 kg·ha^-1^, significantly exceeding that of other germplasm resources. Although its yield was relatively low under LN, it showed superior N agronomic use efficiency, partial factor productivity and utilization index, indicating robust N utilization efficiency. Thus, XN2153–5 could be identified as a low N-, P-, and K-tolerant and nutrient-efficient genotype.

## 4 Discussion

### 4.1 Differences in agronomic traits and nutrient use efficiency of sweetpotato germplasm resources under different nutrient levels at seedling stage

Identifying reliable screening indices and nutrient-efficient germplasm is a cornerstone strategy for advancing sustainable horticulture, particularly for root and tuber crops grown in marginal soils (Nisar et al., 2016). The seedling stage is critical for nutrient acquisition and population establishment (Fillery and McInnes, 1992; Liao et al., 2004; Ahn, 2015), making it essential for evaluating nutrient use efficiency. Our data from the four nutrient regimes revealed that, compared to the control, root length, root fresh weight, and shoot N physiological use efficiency increased under stress, indicating promoted nutrient remobilization and allocation of assimilates to storage organs (Gaju et al., 2011). Concurrently, stomatal conductance, transpiration rate, shoot dry matter accumulation, and shoot nutrient content decreased. This adaptive response may sustain leaf area for photosynthesis while prioritizing root development, consistent with known resource allocation strategies under limitation (Ning et al., 2013; Urban et al., 2021). The coefficient of variation (CV) could assess the suitability of trait indices as screening criteria (Li et al., 2014b). In our study, the CV for most traits increased under stress compared to CK. This indicates that low-nutrient conditions amplify germplasm variability, thereby enabling more accurate screening, aligning with the previous findings (Ge et al., 2021).

### 4.2 Screening of evaluation indicators for N, P and K deficiency tolerance

Reliable evaluation induces are prerequisites for efficient germplasm screening. Nutrient use efficiency, which reflects a plant’s capacity to absorb and utilize nutrients for yield formation (Deng et al., 2020b), is influenced by complex transport and allocation mechanisms (Glass, 1989; Rengel and Damon, 2008). However, evaluation indicators must be crop-specific, aligned with distinct sink organs and growth habits. While cereal crops prioritize grain-related traits, and leafy vegetables emphasize shoot biomass, the evaluation of storage root crops, such as sweetpotato, must integrate both vegetative vigor and root bulking potential. Conventional screening often relies on limited indices or yield alone (Ghimire et al., 2021; Kharel et al., 2011), which may be insufficient for capturing complex stress tolerance. Therefore, a multi-index approach that integrates morphological and physiological traits is essential.

To this end, we employed principal component analysis (PCA) and stepwise regression to distill key indicators from multiple seedling traits under N, P, and K deficiencies. Our integrated PCA and regression analyses successfully identified robust and conserved indicators. At the seedling stage, the first two principal components (PC1 & PC2) exhibited high cumulative contribution rates, primarily representing biomass accumulation and photosynthetic efficiency, respectively, and played a central role in explaining plant responses to all three nutrient stresses. Notably, five seedling traits, including leaf number per plant, shoot fresh weight, root fresh weight, shoot dry weight, and Pn, exhibited consistently high loadings under N, P, and K deficiency stresses. These traits also demonstrated a significant correlation with the comprehensive evaluation value (Y), thereby affirming their universal importance as early screening criteria for nutrient stress tolerance in sweetpotato (Yang, 2023).Regression modeling further validated the predictive power of these traits. For example, under nitrogen deficiency, a model that incorporated main stem length, leaf number per plant, shoot and root fresh weight, shoot dry weight, Pn, and shoot nitrogen physiological use efficiency achieved a high R^2^ value of 0.985. Leaf number per plant and Pn directly influence photosynthetic carbon assimilation and canopy development, forming the essential physiological “source” for economic yield under stress conditions. Similarly, storage root fresh weight, which reflects nutrient uptake and assimilation capacity, serves as a direct proxy for sink strength and future yield potential, demonstrating high loadings under P and K limitations. This methodology is consistent with previous studies that employed similar composite traits for screening nutrient efficiency in crops such as maize and cotton (Zhang et al., 2015; Iqbal et al., 2023).

The identification of a conserved set of five key indicators suggests the potential existence of a core module for early physiological adaptation in sweetpotato, which coordinates source-sink reallocation under nutrient stress. These core traits provide precise phenotypic targets for future multi-omics research aimed at deciphering the shared genetic and molecular underpinnings of nutrient use efficiency in root crops. In our field trails, traits directly linked to economic yield, such as storage root fresh weight, dry weight, nutrient accumulation, and harvest index, were validated as essential metrics for final assessment, confirming that indicators measured at the seedling stage effectively predict field performance outcomes.

### 4.3 Physiological basis linking phenotypic responses to nutrient transport and uptake

The distinct responses in phenotypic traits due to deficiencies in N, P, and K noted during our seedling screening, including greater root length under lower N or low P conditions, a significant decrease in P accumulation in shoots, and enhanced physiological efficiency in K utilization, can be mechanistically associated with the varying mobility of these nutrients within the soil and their specific uptake physiology (Chen and Liao, 2017). Nitrogen, which is highly mobile in the form of nitrate, typically triggers a response aimed at foraging for roots, which correlates with the root elongation we observed under low N stress. This is a frequent strategy used to capture available mobile N sources. Conversely, P presents a unique challenge due to its extremely low mobility resulting from fixation, as evidenced by our findings of considerable root growth coupled with a steep decline in P accumulation in shoots under low P conditions. This indicates that even with morphological changes, the acquisition of P is still significantly limited, thus emphasizing the critical role of rhizosphere modification efficiency beyond just root structure. As for K, which exhibits intermediate mobility, plants frequently improve their internal utilization efficiency when facing deficiency. The data we collected reveal that an enhanced physiological efficiency for shoot K use was a vital adaptive characteristic under low K conditions, aligning with previous research showing that K-efficient genotypes optimize the internal redistribution of K and bolster transporter activity (Rengel and Damon, 2008; Liu et al., 2023; Tang et al., 2014). Therefore, the nutrient-specific and conserved indicators we discovered integrate the physiological bases for acquiring and utilizing nutrients with their differing behaviors in soil.

### 4.4 The two-phase evaluation system for nutrient use efficiency genotypes screening

Sweetpotato’s unique physiology, especially its subterranean development, demands specialized evaluation (Kaupa and Rao, 2014). Notably, the absence of storage root formation during the seedling stage differentiates these evaluations from field-based nutrient use efficiency calculations that utilize storage root yield (Gunes et al., 2006; Hayes et al., 2004). Given that nutrient absorption primarily occurs during the middle to late growth stages (Kaupa and Rao, 2014), sole reliance on seedling-stage traits presents inherent limitations for predicting field performance. To address this methodological gap, we developed a two-phase framework integrating hydroponic screening with yield validation. This novel protocol systematically links seedling phenotyping with nutrient allocation during the storage root formation stage in root crops. This system directly addresses the knowledge gaps regarding root-soil interactions and stage-specific nutrient allocation priorities highlighted in the introduction. By harmonizing seedling-stage adaptability with storage root-bulking requirements, this framework establishes a standardized evaluation system for root crops, effectively bridging the gap between controlled-environment studies and field performance validation.

Our results demonstrated that most of the indices were reduced under low nutrient treatments. However, high nutrient levels can also induce stress in plants. In the field validation experiments, to highlight the plants’ response to nutrient status, the application rate of N (P, K) in HN (HP, HK) treatment was selected to be equal to or slightly higher than the high level among the previously reported optimal N (P, K) application rate (Jia et al., 2016; Shu et al., 2024), thus serving as effective “sufficient nutrient” controls for their respective nutrients, against which the performance under deficiency (LN, LP, or LK) could be accurately assessed. The application rates of other nutrients were correspondingly adjusted to maintain compatibility, and comparisons were made with scenarios where no additional application of the specific nutrient, to improve the efficiency of screening nutrient-efficient germplasm resources. The integrated two-phase approach resolves two critical limitations: root exudate-soil interactions in nutrient acquisition and stage-specific nutrient allocation priorities, effectively integrating hydroponic seedling responses with field-validated nutrient dynamics. Compared to conventional single-stage screening systems (Navakode et al., 2009; Wang et al., 2015), this framework not only reduces evaluation timelines but also improves selection accuracy by establishing biologically consistent linkages between seedling traits (leaf number per plant, shoot/root biomass, photosynthetic rate) and storage root productivity under field conditions.

As both priority breeding materials and functional prototypes for investigating root crop nutrient homeostasis, the nutrient-efficient germplasm identified in this research exemplifies the methodology’s capacity to bridge theoretical understanding with practical crop improvement. From a practical application perspective, the germplasm selected in this study, specifically XN2153–5 and XN1985-7, is particularly well-suited for agricultural production systems characterized by limited fertilizer input or suboptimal soil conditions, such as those found in the hilly drylands of Southwest China. The adoption of these varieties can directly support smallholder farmers by sustaining sweetpotato yields and reducing fertilizer costs associated with chemical fertilizers. This has significant practical implications for supporting sustainable agricultural practices and contributing to rural development goals. This study’s two-phase protocol addresses the critical gaps in prior research by integrating multi-nutrient stress screening with field validation, thereby offering a scalable and efficient model for root crop improvement under nutrient-limited conditions. Although the innovative two-phase evaluation system developed and validated in this study has effectively identified exceptional germplasm resources associated with various nutrient use efficiency-related phenotypes, thereby establishing a robust methodological foundation for subsequent related research. However, it is crucial to recognize that agricultural production is often influenced by the interplay of multiple field factors (Wang et al., 2017; Xu et al., 2022). Future research should prioritize conducting trials across diverse locations and seasons, utilizing larger experimental plots to validate the agronomic performance and adaptability of the outstanding germplasm identified herein. This approach will yield more reliable recommendations for the sustainable production of sweetpotato in low-fertility environments.

## 5 Conclusion

This study established a two-phase protocol combining hydroponic seedling screening with field validation. This approach identified key evaluation indices and nutrient-efficient sweetpotato genotypes. We identified robust criteria for assessing nutrient use efficiency under limitations of nitrogen, phosphorus, and potassium and characterized elite germplasms that consistently performed well across both developmental stages under nutrient stress. Consequently, this work not only provides practical tools for efficient germplasm screening but also offers vital genetic resources for future breeding programs and mechanistic studies aimed at enhancing sustainable sweetpotato production in low-fertility agroecosystems.

## Data Availability

The original contributions presented in the study are included in the article/**Supplementary Material**. Further inquiries can be directed to the corresponding authors.
