# Supplementary material for: A two-phase evaluation system integrating hydroponic and field screening identifies nutrient-efficient sweetpotato (Ipomoea batatas (L.) Lam.) germplasm
Source: Front Plant Sci. 2026 Feb 25;17:1780928. doi: 10.3389/fpls.2026.1780928 (PMC12975940; doi:10.3389/fpls.2026.1780928)
Supplement: Supplementary file 1 [file Table1.docx]

Table S1 List of sweet potato germplasm resources used in the study

| No. | Germplasm | primary screening | Secondary screening | No. | Germplasm | primary screening | Secondary screening |
| --- | --- | --- | --- | --- | --- | --- | --- |
| 1 | XN2188-2 | √ |  | 19 | XN2102-2 | √ |  |
| 2 | XN2154-1 | √ | √ | 20 | XN2150-1 | √ |  |
| 3 | XN2126-8 | √ |  | 21 | XN2138-1 | √ |  |
| 4 | XN2160-1 | √ |  | 22 | XN21122-1 | √ |  |
| 5 | XN2188-3 | √ |  | 23 | XN2126-9 | √ |  |
| 6 | XN2155-1 | √ | √ | 24 | XN2149-1 | √ |  |
| 7 | Ningzishu No.1 | √ |  | 25 | XN1944-4 | √ |  |
| 8 | XN2153-5 | √ | √ | 26 | XN1756-2 | √ |  |
| 9 | XN2153-1 | √ | √ | 27 | XN1878-7 | √ | √ |
| 10 | XN2134-1 | √ |  | 28 | XN1985-7 | √ | √ |
| 11 | XN2258 | √ |  | 29 | XN2030-11 | √ |  |
| 12 | XN2155-7 | √ |  | 30 | XN1746-1 | √ |  |
| 13 | XN2124-2 | √ |  | 31 | XN1915-4 | √ |  |
| 14 | XN2152-3 | √ |  | 32 | XN17104-132 | √ | √ |
| 15 | XN2141-3 | √ | √ | 33 | XN1867-3 | √ |  |
| 16 | Shangshu 19 | √ |  | 34 | XN1764-7 | √ |  |
| 17 | XN21110-7 | √ |  | 35 | XN21110-1 | √ |  |
| 18 | XN2116-2 | √ |  |  | subtotal | 35 copies | 8 copies |

Table S2 Hydroponic solution used in four nutrient treatments

| Fertilizer Treatment | Nutrient components and content | |
| --- | --- | --- |
|  | macronutrients | micronutrients |
| CK | Ca(NO_3_)_2_·4H_2_O：945 mg L^-1^  KNO_3_：607 mg L^-1^  NH_4_(H_2_PO_4_)：115 mg L^-1^  MgSO_4_·7H_2_O：493 mg L^-1^ | Na_2_FeEDTA：40 mg L^-1^  H_3_BO_3_：2.86 mg L^-1^  MnSO_4_·4H_2_O：2.13 mg L^-1^  ZnSO_4_·7H₂O：0.22 mg L^-1^  CuSO_4_·5H_2_O：0.08 mg L^-1^  (NH_4_)_6_Mo_7_O_24_·4H_2_O：0.02 mg L^-1^ |
| LN | CaCl_2_：350 mg L^-1^  KCl：200 mg L^-1^  MgSO_4_·7H_2_O：493 mg L^-1^  KH_2_PO_4_：250 mg L^-1^ |  |
| LP | Ca(NO_3_)_2_：940 mg L^-1^  KNO_3_：700 mg L^-1^  MgSO_4_·7H_2_O：493 mg L^-1^ |  |
| LK | Ca(NO_3_)_2_：946 mg L^-1^  NH_4_(H_2_PO_4_)：115 mg L^-1^  MgSO_4_·7H_2_O：493 mg L^-1^ |  |

Table S3 Soil nutrient conditions of the experimental plot at a depth of 0-20 cm

| pH | Total nitrogen  (g kg^-1^) | Total phosphorus  (g kg^-1^) | Total potassium  (g kg^-1^) | Soil organic matter  (g kg^-1^) | Available potassium  (mg kg^-1^) | Available phosphorus  (mg kg^-1^) | Alkaline nitrogen  (mg kg^-1^) |
| --- | --- | --- | --- | --- | --- | --- | --- |
| 8.29 | 11.17 | 1.29 | 27.54 | 14 | 113.01 | 39.97 | 105.71 |

Table S4 Fertilizer treatments in the field trail

| Experimental  treatments | Nitrogen  (kg N ha^-1^) | Phosphorus  (kg P_2_O_5_ ha^-1^) | Potassium  (kg K_2_O ha^-1^) |
| --- | --- | --- | --- |
| Low nitrogen (LN) | 0 | 90 | 220 |
| High nitrogen (HN) | 180 | 90 | 220 |
| Low phosphorus (LP) | 155 | 0 | 220 |
| High phosphorus (HP) | 155 | 120 | 220 |
| Low potassium (LK) | 155 | 90 | 0 |
| High potassium (HK) | 155 | 90 | 240 |

Table S5 Classification of sweet potato germplasm resources under low nitrogen (N), phosphorus (P) and potassium (K) conditions at seedling stage

| Methodologies | Typology | Number of germplasms | Germplasms |
| --- | --- | --- | --- |
| Clustering of low-N/P/K stress tolerance indices at the seedling stage | Low N-, P- and K- tolerant type | 13 | XN2126-8, XN2160-1, XN2188-3, XN2153-5, XN2134-1, XN2152-3, XN2141-3, XN2116-2, XN2102-2, XN2150-1, XN2126-9, XN1944-4, XN1985-7 |
|  | Low N-, P- and K-intolerant type | 1 | XN2153-1 |
| Comprehensive evaluation value of nutrient use efficiency at the seedling stage | N, P and K efficient-efficient type | 4 | XN2188-3, XN2153-5, XN21110-7, XN1944-4 |
|  | N, P and K inefficient-inefficient type | 5 | XN2154-1, Ningzishu No.1, XN2153-1, XN2155-7, XN21122-1 |
| Clustering of low N stress tolerance indices at the seedling Stage and comprehensive evaluation value of N use efficiency | Low N-tolerant and N-efficient type | 3 | XN1746-1, XN1915-4, XN21110-1 |
|  | Low N-sensitive and N-inefficient type | 5 | XN2188-3, XN2153-5, XN2141-3, XN2150-1, XN2149-1, XN17104-32, XN1878-7 |
| Clustering of low P stress tolerance indices at the seedling stage and comprehensive evaluation value of P use efficiency | Low P-tolerant and P-efficient type | 3 | XN1944-4, XN1985-7, XN2030-11, XN17104-32, XN1878-7 |
|  | Low P-sensitive and P-inefficient type | 5 | XN2154-1, XN2155-1, Ningzishu No.1, XN2153-1, XN2155-7 |
| Clustering of low K stress tolerance indices at the seedling stage and comprehensive evaluation value of K use efficiency | Low K-tolerant and K-efficient type | 4 | Shangshu 19, XN1746-1, XN1915-4, XN1764-7 |
|  | Low K-sensitive and K-inefficient type | 5 | XN2188-3, XN2153-5, XN2134-1, XN2141-3, XN21110-7 |

Table S6 Agronomic traits and nitrogen dynamics of sweet potato germplasm under nitrogen treatments

| Indices | High nitrogen treatments | | | Low nitrogen treatments | | | Comparison of high nitrogen and low nitrogen | |
| --- | --- | --- | --- | --- | --- | --- | --- | --- |
|  | Scope | Average value | CV (%) | Scope | Average value | CV (%) | Reduction rate (%) | Increase of CV under low nitrogen |
| Storage root fresh weight (g) | 157.92~372.71 | 252.75 | 32.64 | 125~286.67 | 189.99 | 26.85 | 24.83 | -5.79 |
| Storage root dry weight(g) | 38.92~92.44 | 66.40 | 30.98 | 31.49~74.84 | 54.07 | 25.38 | 18.57 | -5.60 |
| Shoot dry weight (g) | 15.69 to 192.35 | 63.95 | 84.86 | 20.6~115.3 | 51.41 | 58.94 | 19.61 | -25.92 |
| Whole plant dry weight (g) | 54.6~284.79 | 130.36 | 51.72 | 61.95~170.51 | 105.48 | 29.28 | 19.09 | -22.44 |
| Root-shoot ratio | 0.48~2.48 | 1.43 | 51.30 | 0.48~3.63 | 1.42 | 73.68 | 0.70 | 22.38 |
| Shoot N content (g g⁻^1^) | 17.8~26.18 | 22.33 | 13.04 | 15.71~31.42 | 20.35 | 25.35 | 8.87 | 12.31 |
| Storage root N content (g g⁻^1^) | 3.93~7.33 | 5.69 | 20.78 | 1.83~6.02 | 4.06 | 40.40 | 28.65 | 19.62 |
| Total N content (g g⁻^1^) | 23.04~32.07 | 28.03 | 11.89 | 17.54~36 | 24.41 | 24.58 | 12.91 | 12.69 |
| Shoot N accumulation value (g plant⁻^1^) | 0.38~3.42 | 1.34 | 68.72 | 0.32~3.62 | 1.18 | 91.02 | 11.94 | 22.30 |
| Storage root N accumulation value  (g plant⁻^1^) | 0.22~0.55 | 0.37 | 29.37 | 0.07~0.42 | 0.22 | 50.45 | 40.54 | 21.08 |
| Total N accumulation value  (g plant⁻^1^) | 0.67~3.91 | 1.71 | 57.73 | 0.46~3.88 | 1.40 | 78.11 | 18.13 | 20.38 |
| N harvest index | 0.12~0.43 | 0.25 | 36.94 | 0.07~0.42 | 0.20 | 58.96 | 20.00 | 22.02 |
| Plant N uptake efficiency (%) | 16.43~96.54 | 42.13 | 57.73 | / | / | / | / | / |
| Storage root N utilization efficiency  (kg kg⁻^1^) | 23.65~66.97 | 44.68 | 34.40 | 14.24~162.42 | 58.19 | 78.84 | -30.24 | 44.44 |
| Plant N utilization efficiency rate  (kg kg⁻^1^) | 61.12~94.45 | 79.11 | 13.83 | 44~207.13 | 98.67 | 49.87 | -24.73 | 36.04 |

Table S7 Agronomic traits and phosphorus dynamics of sweet potato germplasm under phosphorus treatments

| Indices | High phosphorus treatment | | | Low phosphorus treatment | | | Comparison of high phosphorus and low phosphorus | |
| --- | --- | --- | --- | --- | --- | --- | --- | --- |
|  | Scope | Average  value | Reduction rate (%) | Increase of CV under low phosphorus | Average value | CV (%) | Reduction rate (%) | Increase of CV under low phosphorus |
| Storage root fresh weight (g) | 139.91 to 412.5 | 261.38 | 34.96 | 84.17~310 | 194.73 | 33.07 | 25.50 | -1.89 |
| Storage root dry weight (g) | 40.55~101.49 | 70.93 | 31.24 | 28.12~98.23 | 54.32 | 38.64 | 23.42 | 7.40 |
| Shoot dry weight (g) | 20.29~126.93 | 54.68 | 76.16 | 22.85~137.96 | 50.75 | 73.29 | 7.19 | -2.87 |
| Whole plant dry weight (g) | 60.84~217.35 | 125.61 | 42.94 | 64.59~181.85 | 105.07 | 40.07 | 16.35 | -2.87 |
| Root-shoot ratio | 0.61~4.74 | 1.88 | 68.02 | 0.32~2.19 | 1.38 | 49.19 | 26.60 | -18.83 |
| Shoot phosphorus content (g g^-1^) | 3.01~4.52 | 3.43 | 15.73 | 2.64~4.81 | 3.40 | 20.60 | 0.87 | 4.87 |
| Storage root phosphorus content (g g^-1^) | 1.62~2.36 | 1.87 | 11.97 | 1.47~2.54 | 1.90 | 17.19 | -1.60 | 5.22 |
| Total phosphorus content (g g^-1^) | 4.67~6.32 | 5.31 | 9.74 | 4.6~6.42 | 5.30 | 10.88 | 0.19 | 1.14 |
| Shoot phosphorus accumulation  (g plant^-1^) | 0.06~0.57 | 0.20 | 93.71 | 0.06~0.5 | 0.18 | 79.71 | 10.00 | -14.00 |
| Storage root phosphorus accumulation  (g plant^-1^) | 0.09~0.19 | 0.13 | 29.92 | 0.06~0.14 | 0.10 | 31.23 | 23.08 | 1.31 |
| Total phosphorus accumulation  (g plant^-1^) | 0.16~0.74 | 0.34 | 61.91 | 0.16~0.59 | 0.28 | 51.29 | 17.65 | -10.62 |
| Phosphorus harvest index | 0.22~0.71 | 0.47 | 36.61 | 0.15~0.66 | 0.41 | 41.91 | 12.77 | 5.30 |
| Plant phosphorus uptake efficiency (%) | 5.80~27.29 | 12.42 | 61.91 | / | / | / | / | / |
| Storage root phosphorus utilization efficiency (kg kg^-1^) | 120.82~403.17 | 251.88 | 37.29 | 74.77~313.62 | 218.61 | 37.81 | 13.21 | 0.52 |
| Plant phosphorus utilization efficiency  (kg kg^-1^) | 295.07~488.16 | 404.82 | 16.87 | 308.03~459.7 | 390.75 | 15.58 | 3.48 | -1.29 |

Table S8 Agronomic traits and potassium dynamics of sweet potato germplasm under potassium treatments

| Indices | High potassium treatment | | | Low potassium treatment | | | Comparison of high potassium and low potassium | |
| --- | --- | --- | --- | --- | --- | --- | --- | --- |
|  | Scope | Average value | CV (%) | Scope | Average value | CV (%) | Reduction rate (%) | Increase of CV under LK |
| Storage root fresh weight (g) | 145.58~583.38 | 292.82 | 47.32 | 119.88~461.25 | 252.13 | 41.43 | 13.90 | -5.89 |
| Storage root dry weight (g) | 40.53~171.18 | 85.15 | 50.92 | 30.54 to 121.2 | 65.02 | 43.48 | 23.64 | -7.44 |
| Shoot dry weight (g) | 16.67~174.47 | 77.53 | 73.20 | 15.65~179.46 | 77.79 | 74.24 | -0.34 | 1.04 |
| Whole plant dry weight (g) | 57.2 to 252.5 | 162.69 | 43.60 | 77.07~263.37 | 142.81 | 51.97 | 12.22 | 8.37 |
| Root-shoot ratio | 0.45~3.43 | 1.63 | 67.02 | 0.45~3.93 | 1.33 | 88.51 | 18.40 | 21.49 |
| Shoot potassium content (g g^-1^) | 46.9~64.55 | 54.76 | 11.74 | 40.08~65.1 | 54.19 | 16.19 | 1.04 | 4.45 |
| Root potassium content (g g^-1^) | 17.16~26.79 | 22.17 | 18.60 | 12.79~29.81 | 21.62 | 24.01 | 2.48 | 5.41 |
| Total potassium content (g g^-1^) | 64.7~91.34 | 76.94 | 12.00 | 61.74~90.63 | 75.80 | 12.69 | 1.48 | 0.69 |
| Shoot potassium accumulation value  (g plant^-1^) | 0.84~10.31 | 4.27 | 73.49 | 0.81~11.46 | 4.50 | 84.48 | -5.39 | 10.99 |
| Storage root potassium accumulation value (g plant^-1^) | 0.96~3.91 | 1.85 | 51.15 | 0.59~2.14 | 1.36 | 39.06 | 26.49 | -12.09 |
| Total potassium accumulation value  (g plant^-1^) | 1.8~12.31 | 6.12 | 54.72 | 2.4 to 12.54 | 5.86 | 67.17 | 4.25 | 12.45 |
| Potassium harvest index | 0.16~0.56 | 0.36 | 44.83 | 0.09~0.66 | 0.31 | 62.14 | 13.89 | 17.31 |
| Plant potassium uptake efficiency (%) | 33.36~228.12 | 113.30 | 54.72 | / | / | / | / | / |
| Storage root potassium utilization efficiency (kg kg^-1^) | 6.34~26.27 | 16.69 | 48.57 | 6.05~25.56 | 14.09 | 53.55 | 15.58 | 4.98 |
| Plant potassium utilization efficiency  (kg kg^-1^) | 20.19~37.21 | 28.57 | 21.15 | 19.53~36.01 | 26.83 | 21.02 | 6.09 | -0.13 |

Table S9 Summary of nutrient deficiency tolerance and nutrient efficiency of germplasms in two phases

| Germplasms | Hydroponic screening | | | | | | Field trail | | |
| --- | --- | --- | --- | --- | --- | --- | --- | --- | --- |
|  | Nutrient deficiency tolerance^*^ | | | Nutrient efficiency^**^ | | | Nutrient efficiency | | |
|  | Low-nitrogen | Low-phosphorus | Low-potassium | nitrogen | phosphorus | potassium | nitrogen | phosphorus | potassium |
| XN2153-1 | intolerant | intolerant | intolerant | Inefficient-inefficient | Inefficient-inefficient | Inefficient-inefficient | Inefficient-inefficient | Efficient-inefficient | Inefficient-inefficient |
| XN2153-5 | tolerant | tolerant | tolerant | Efficient-efficient | Efficient-efficient | Efficient-efficient | Inefficient-efficient | Efficient-efficient | Efficient-efficient |
| XN1985-7 | tolerant | tolerant | tolerant | Efficient-efficient | Efficient-efficient | Efficient-efficient | Efficient-efficient | Inefficient-efficient | Efficient-inefficient |
| XN17104-132 | intolerant | tolerant | tolerant | Inefficient-efficient | Inefficient-efficient | Efficient-efficient | Inefficient-inefficient | Inefficient-inefficient | Efficient-efficient |
| XN2154-1 | intolerant | tolerant | tolerant | Inefficient-inefficient | Inefficient-inefficient | Inefficient-inefficient | Efficient-inefficient | Inefficient-inefficient | Inefficient-inefficient |
| XN2155-1 | intolerant | tolerant | tolerant | Inefficient-inefficient | Efficient-inefficient | Inefficient-inefficient | Efficient-inefficient | Efficient-inefficient | Inefficient-inefficient |
| XN1878-7 | intolerant | tolerant | tolerant | Inefficient-efficient | Inefficient-efficient | Efficient-efficient | Efficient-efficient | Inefficient-efficient | Inefficient-inefficient |
| XN2141-3 | tolerant | tolerant | tolerant | Efficient-efficient | Efficient-efficient | Inefficient-efficient | Inefficient-efficient | Efficient-efficient | Efficient-efficient |

The low nitrogen / phosphorus / potassium tolerance and nutrient efficiency type of germplasms in hydroponic screening were derived from the data shown in Figure 1 and Figure 2, respectively.
